# HER3-Receptor-Mediated STAT3 Activation Plays a Central Role in Adaptive Resistance toward Vemurafenib in Melanoma

**DOI:** 10.3390/cancers12123761

**Published:** 2020-12-14

**Authors:** Laura Hüser, Marianthi-Maria Kokkaleniou, Karol Granados, Jennifer Dworacek, Aniello Federico, Marlene Vierthaler, Daniel Novak, Ihor Arkhypov, Thomas Hielscher, Viktor Umansky, Peter Altevogt, Jochen Utikal

**Affiliations:** 1 Skin Cancer Unit, German Cancer Research Center (DKFZ), 69120 Heidelberg, Germany; maria.kokkaleniou@upf.edu (M.-M.K.); karolandrea.granados@ucr.ac.cr (K.G.); j.dworacek@dkfz-heidelberg.de (J.D.); a.federico@kitz-heidelberg.de (A.F.); m.vierthaler@dkfz-heidelberg.de (M.V.); d.novak@Dkfz-Heidelberg.de (D.N.); Ihor.Arkhypov@medma.uni-heidelberg.de (I.A.); V.Umansky@dkfz-heidelberg.de (V.U.); p.altevogt@dkfz-heidelberg.de (P.A.); j.utikal@Dkfz-Heidelberg.de (J.U.); 2 Department of Dermatology, Venereology and Allergology, University Medical Center Mannheim, Ruprecht-Karl University of Heidelberg, 68167 Mannheim, Germany; 3 Biostatistics, German Cancer Research Center (DKFZ), 69120 Heidelberg, Germany; t.hielscher@Dkfz-Heidelberg.de

**Keywords:** STAT3, HER3, BRAF inhibitor, adaptive resistance, melanoma

## Abstract

**Simple Summary:**

The major obstacle for the long-term success of targeted therapies in melanoma is the occurrence of resistance. Here, we present a new mechanism of targeted therapy resistance in melanoma where the treatment with the BRAF inhibitor vemurafenib causes an increased activation of HER3 via shed ligands. This is followed by an activation of STAT3 via HER3 and results in the expression of the STAT3 target gene *SOX2*. Pharmacological inhibition of HERs sensitizes melanoma cells toward vemurafenib treatment. Thus, blocking HER family members and especially HER3 in addition to targeted therapy treatment might prevent the occurrence of resistance.

**Abstract:**

Melanoma is an aggressive form of skin cancer that is often characterized by activating mutations in the Mitogen-Activated Protein (MAP) kinase pathway, causing hyperproliferation of the cancer cells. Thus, inhibitors targeting this pathway were developed. These inhibitors are initially very effective, but the occurrence of resistance eventually leads to a failure of the therapy and is the major obstacle for clinical success. Therefore, investigating the mechanisms causing resistance and discovering ways to overcome them is essential for the success of therapy. Here, we observed that treatment of melanoma cells with the B-Raf Proto-Oncogene, Serine/Threonine Kinase (BRAF) inhibitor vemurafenib caused an increased cell surface expression and activation of human epidermal growth factor receptor 3 (HER3) by shed ligands. HER3 promoted the activation of signal transducer and activator of transcription 3 (STAT3) resulting in upregulation of the STAT3 target gene SRY-Box Transcription Factor 2 (*SOX2*) and survival of the cancer cells. Pharmacological blocking of HER led to a diminished STAT3 activation and increased sensitivity toward vemurafenib. Moreover, HER blocking sensitized vemurafenib-resistant cells to drug treatment. We conclude that the inhibition of the STAT3 upstream regulator HER might help to overcome melanoma therapy resistance toward targeted therapies.

## 1. Introduction

Melanoma, a skin cancer that arises from melanocytes, causes 80% of skin cancer-related deaths demonstrating the aggressiveness of this disease [1]. Nowadays, two major approaches are used in clinics for advanced melanoma therapy: immunotherapies and targeted therapies. Since melanoma is often characterized by mutations in B-Raf Proto-Oncogene, Serine/Threonine Kinase (*BRAF*)and NRAS Proto-Oncogene (*NRAS*), causing a hyper-activation of the BRAF–MEK–ERK pathway leading to the proliferation and survival of cancer cells, small molecules targeting this pathway were developed. In clinical practice, BRAF inhibitors such as vemurafenib (vem) are combined with MEK inhibitors such as cobimetinib. Targeted therapies are powerful in the beginning of the treatment, but the occurrence of resistance significantly diminishes the clinical success [2]. Three different forms of resistance toward vem are distinguished. First, intrinsic resistance where the tumor cells do not respond at all to the treatment from the beginning. This is the case in around 15% of melanoma patients [3]. Adaptive resistance, which occurs shortly after onset of treatment, and acquired resistance, which develops over continuous BRAF inhibitor treatment [4]. Since resistance limits the success of the therapy, understanding the molecular mechanisms that underlie resistance and finding ways to counteract them is of high importance for the clinical success of the therapy.

Signal transducer and activator of transcription 3 (STAT3) was shown to play an important role in the development of cancer cell resistance to therapies [5,6,7,8]. STAT3 is a transcription factor, which becomes activated in response to cascades initiated by several cytokines and growth factors [9]. Upon activation, STAT3 is phosphorylated at Ser727 and Tyr705 or acetylated at Lys685 resulting in its dimerization and translocation to the nucleus [10,11]. Furthermore, STAT3 palmitoylation was recently shown to promote the translocation to the nucleus [12]. In the nucleus, STAT3 augments the expression of several genes involved in cell growth, apoptosis, and cell differentiation [13].

STAT3 can be phosphorylated following the activation of receptor tyrosine kinases (RTKs) such as human epidermal growth factor receptor 3 (HER3). HER3 is a member of the HER family consisting of four members (HER1–4). The different family members interact with each other, i.e., HER3, which lacks intrinsic kinase activity, can form heterodimers with HER1, HER2, and HER4 [14]. RTKs such as HERs get activated by ligand binding or by accumulation of RTKs at the cell surface in a ligand-independent way [15]. Due to an important role of HER3 in cancer progression, several therapies targeting HER3 were developed [16]. Additionally, HER3 was shown to play a role in resistance toward targeted therapies [17,18,19].

Here, we show, that HER3 is activated in melanoma cells upon vem treatment via shed ligands. HER3-containing receptors then promote the activation of STAT3, which can be pharmacologically blocked by inhibitors targeting HER family members. The elevated STAT3 expression causes an increase in the expression of SRY-Box Transcription Factor 2 (*SOX2*), which is an important factor in melanoma cell resistance as shown before [8]. This finding demonstrates the central role of STAT3 in adaptive resistance of melanoma cells toward vem. Moreover, the blockade of HER receptor signaling sensitizes the cells toward vem treatment. Therefore, targeting the HER family as an upstream regulator of STAT3, in addition to BRAF and MEK inhibition, might be a promising way to counteract the resistance.

## 2. Results

**Figure 1:**
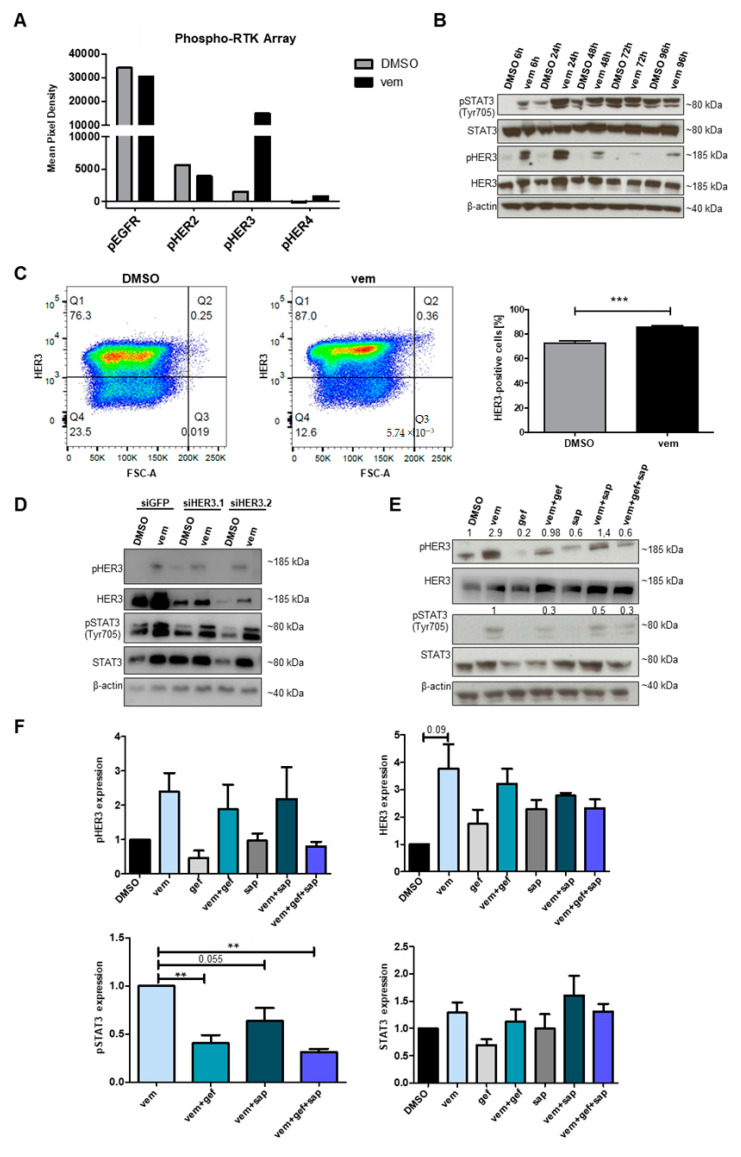
Human epidermal growth factor receptor 3 (HER3) activation is responsible for vemurafenib (vem)-induced signal transducer ad activator of transcription 3 (STAT3) activation. (**A**) Phospho-RTK (receptor tyrosine kinase) array performed on A375 cells after 24 h of vem (3 µM) treatment. The expression level displayed as mean pixel density of phosphorylated EGFR, HER2–4 is shown. (**B**) Western blot, demonstrating that after 24 h of vem (3 µM) treatment a maximum expression of phosphorylated HER3 and STAT3 is reached in A375 cells. (**C**) Dot plots showing an increased percentage of HER3-positive cells after vem (3 µM) treatment of A375 cells. (**D**) An siRNA-mediated knockdown (KD) of HER3 in A375 cells can only mildly prevent HER3 and STAT3 activation. (**E**) Pharmacological inhibition of HER receptors with gefitinib (gef) (10 µM), sapitinib (sap) (10 µM), or a combination of both can diminish HER3 and STAT3 activation in A375 cells. (**F**) Band intensity measurements of the Western blot shown in E as well as the biological replicates of the experiment are shown as relative expression values normalized to the loading control. Only statistically significant values are indicated above the bar graph (*p* < 0.01 **; *p* < 0.001 ***).

To investigate the molecular mechanisms promoting the escape of melanoma cells from targeted therapies, we examined the phosphorylation/activation status of different RTKs following vem treatment. We performed a phospho-RTK array with A375 melanoma cells treated for 24 h with vem or DMSO as a control. Only HER3 was highly phosphorylated in vem-treated cells, whereas the phosphorylation status of the other HER family members was not changed (Figure 1A, Supplementary Figure S1A). Next, we analyzed the phosphorylation of HER3 and STAT3 on the protein level after the treatment of A375 and SK Mel 28 melanoma cells with vem or DMSO at different time points (Figure 1B, Supplementary Figure S1B,C). In A375 cells, HER3 as well as STAT3 showed the strongest phosphorylation after 24 h of vem treatment (Figure 1B). At this time point, the STAT3 targets, CD24 and SOX2, that we identified before [8] were strongly upregulated (Supplementary Figure S1B). In SK Mel 28 cells, HER3 and STAT3 were already strongly phosphorylated after 6 h of treatment. Moreover, STAT3 and HER3 activation and the expression of *SOX2* all associated with increased resistance were also increased after treatment with the commonly used combination of vemurafenib and cobimetinib or cobimetinib alone. In addition, treatment with trametinib increased STAT3 activation and expression of *SOX2* (Supplementary Figure S1D). Furthermore, we observed a significant increase in HER3-positive cells after vem treatment in three different melanoma cell lines by flow cytometry (Figure 1C, Supplementary Figure S1F,G).

**Figure 2:**
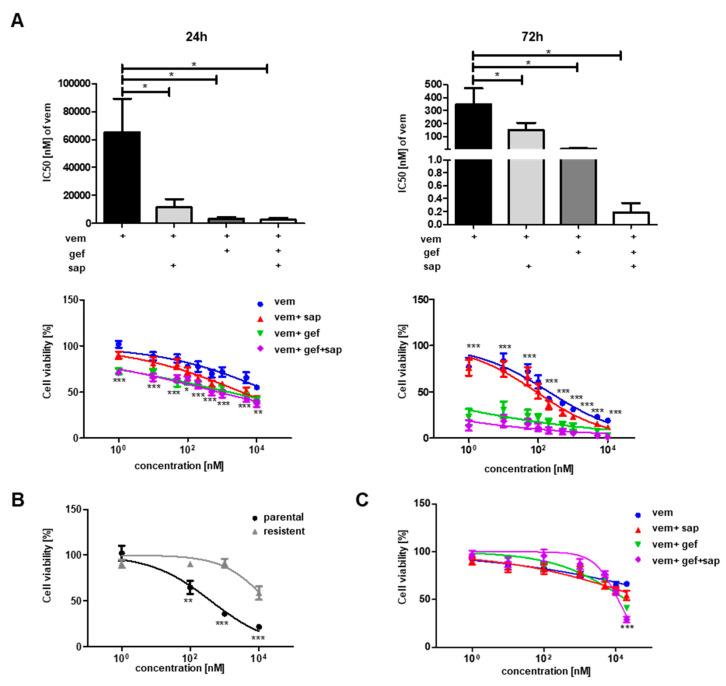
Inhibiting HER3 sensitizes melanoma cells toward vem treatment. (**A**) A375 cells were treated with increasing concentrations of vem (as indicated) alone or in combination with 10 µM sap/gef as indicated. An alamarBlue viability assay was performed to determine the dose–response curves and IC_50_ values for vem alone as well as in combination with gef and sap after 24 h of treatment. Each combination was statistically analyzed in comparison to vem alone. The significance in the dose–response curve refers to the comparison of vem vs. vem + gef + sap. (**B**) Dose–response curve of A375 parental vs. A375-res cells after 72 h of vem treatment in the indicated concentrations. (**C**) Dose–response curves of A375-res cells treated for 24 h with vem as well as with the combinations of vem, gef, and sap. The drugs were mixed in the same concentrations of each drug. The concentration of each drug is indicated on the x-axis. Significance indication refers to vem vs. vem + gef + sap. Thus, HER inhibition sensitizes vem-resistant cells (*p* < 0.05 *; *p* < 0.01 **; *p* < 0.001 ***).

To further examine whether vem-induced STAT3 activation was dependent on HER3, an siRNA-mediated knockdown (KD) was performed (Figure 1D, Supplementary Figure S1E). Although the KD of HER3 decreased the expression level by approximately 80%, the remaining HER3 was still enough to be phosphorylated and thus STAT3 was phosphorylated as well (Figure 1D). For this reason, a pharmacological approach was applied to block HER3 phosphorylation. Gefitinib (gef), an inhibitor of Epidermal Growth Factor Receptor (EGFR) as well as HER2, 3, and 4, and sapitinib (sap), a more specific inhibitor of HER2 and 3, were used alone or in combination with vem to treat A375 cells for 24 h. Each inhibitor diminished the vem-induced phosphorylation of HER3 and STAT3 (Figure 1E,F). Importantly, the combination of gef and sap showed the strongest effect on STAT3 phosphorylation (Figure 1E,F). To study the effect of gef and sap on the sensitivity of melanoma cells toward vem, we performed a viability assay and determined the dose–response curves and the half maximal inhibitory concentration (IC_50_) values. Each inhibitor alone or in combination rendered A375 (Figure 2A) and SK Mel 28 cells (Supplementary Figure S2A) more sensitive toward vem treatment. Moreover, A375-res cells, which are resistant to vem (Figure 2B), were treated with sap and gef in addition to vem. The addition of HER inhibitors sensitized the resistant cells toward vem treatment (Figure 2C). However, HER3 was not detected at the cell surface of resistant cells although phosphorylated HER3 was still detectable (data not shown). Thus, the effects observed here reflect most likely a blockage of HER family members rather than being HER3 specific.

To test whether the drugs acted synergistically or additive, A375 and A375-res cells were treated with all single drugs as well as with the combinations (Supplementary Figure S2B,C). The Loewe additivity method and the associated combination index (CI) were used to calculate whether the drugs acted synergistically or additively. The calculation showed that according to the CI (upper limit of confidence interval) only the highest concentration (20 µM) showed that the drugs work synergistically, while in lower concentrations the upper confidence limit of the CI was not below 1 indicating additivity (Supplementary Table S1). It can be concluded that pharmacological inhibition of HER impaired the resistance toward vem.

**Figure 3:**
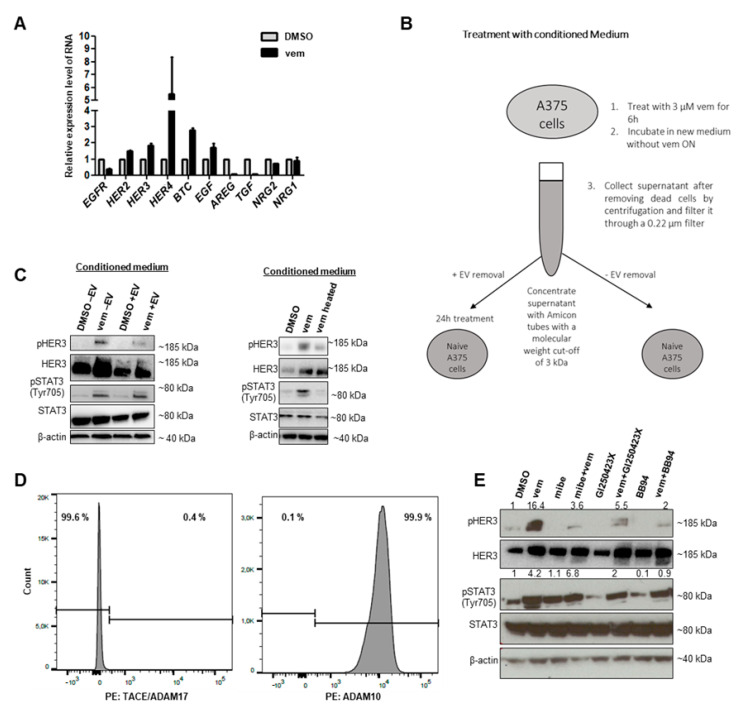
HER3 activation is caused by soluble shed ligands. (**A**) Quantification of the expression of *HER* family members and their ligands relative to 18S by qPCR in A375 cells upon DMSO or vem (3 µM) treatment (24 h). (**B**) Schematic figure of the treatment of naïve A375 cells with conditioned medium in order to investigate the mechanism of HER3 activation. (**C**) Left: Conditioned medium produced by A375 cells pre-treated with DMSO/vem (3 µM) was used to treat naïve cells. Vem-conditioned medium is able to activate HER3 and consequently STAT3, while the removal of extracellular vesicles (EVs) by ultracentrifugation only marginally reduced the effect. Right: The stimulatory component in the supernatant is heat sensitive (boiling for 5 min at 95 °C), showing that the effect is not caused by residual vem. (**D**) Flow cytometry analysis showing no expression of ADAM (a disintegrin and metalloprotease) domain 17 TACE/ADAM17 but high expression of ADAM10 in A375 cells. (**E**) A375 cells were treated for 24 h with DMSO, vem (3 µM), mibefradil (7 µM), GI254023x (5 µM), BB94 (10 µM) as indicated. The sheddase inhibitors GI254023x and BB94 can diminish HER3 and STAT3 activation. Densitometry was normalized to DMSO relative to β-actin.

Next, we analyzed how vem induced the activation of HER3. Therefore, the expression of *HER* family members and their ligands was analyzed by qPCR in different melanoma cell lines (Figure 3A and Supplementary Figure S3A). The expression of *HER3* and *4* was increased upon vem treatment. Among the ligands, betacellulin (*BTC*) and epidermal growth factor (*EGF*) showed slightly stronger expression. In contrast, the expression of amphiregulin (*AREG*) and transforming growth factor (*TGF*) was decreased.

Then, we tested whether recombinant neuregulin 1 and 2 (NRG1 and NRG2), the most known ligands of HER3, or BTC could stimulate HER3 and STAT3 phosphorylation. Neither the treatment with recombinant BTC nor that with NRG1 and NRG2 altered the activation of HER3 and STAT3 in A375 melanoma cells. In contrast, recombinant NRG1 was able to activate HER3 in MCF7 breast cancer cells (data not shown).

To test whether vem-treated cells secreted a soluble ligand able to trigger HER3 and STAT3 activation, we used another approach (outlined in Figure 3B). A375 cells were exposed to DMSO or vem for 6 h. Subsequently, the cells were washed twice with fresh medium followed by incubation in fresh medium overnight. The overnight conditioned medium (CM) was collected, centrifuged to remove dead cells, and then filtered through a 0.22 µm filter. To ensure that no residual vem remained and no potential ligand was lost, we performed size fractionation using Amicon filter units with a defined cutoff of 3 kDa. Only molecules with a molecular weight above 3 kDa (vem has a size of 490 Da) were concentrated and resuspended in fresh medium. In order to investigate a possible role of extracellular vesicles (EVs) in HER and STAT3 activation, the vesicles were removed from the supernatant by ultracentrifugation at 100,000× *g* for 2 h. Moreover, a part of the CM was boiled to destroy activity. The activation of HER3 and STAT3 was analyzed in naïve A375 cells upon their incubation for 24 h with the boiled or EV-depleted or non-treated CM by Western blot.

We found that the vem-induced HER3 and STAT3 activation was mediated by a compound with a size larger than 3 kDa and sensitivity to heat. Interestingly, both EV-depleted and full CM exerted comparable effects suggesting that EVs did not contribute to HER3 and STAT3 activation (Figure 3C).

Since most ligands of the HER family need to be cleaved from the cell surface by sheddases [20], we analyzed the cell surface expression of the sheddases ADAM (a disintegrin and metalloprotease) domain 17 (TACE/ADAM17) and ADAM Metallopeptidase Domain 10 (ADAM10) on melanoma cells by flow cytometry. A375 as well as SK Mel 28 cells showed high expression of ADAM10, while TACE was not detected (Figure 3D, Supplementary Figure S3B). The treatment of cells with the ADAM10 inhibitor GI250423x and with the broad-spectrum matrix metalloproteinase inhibitor BB94 diminished the vem-induced activation of HER3 and STAT3 (Figure 3E). Interestingly, the T-type calcium channel blocker mibefradil, which was previously shown to restore the sensitivity of resistant melanoma cells to BRAF and MEK inhibitors [21], was also able to reduce vem-induced HER3 and STAT3 activation. Hence, a vem-induced ligand shedding process stimulated HER3 and STAT3 activation.

**Figure 4:**
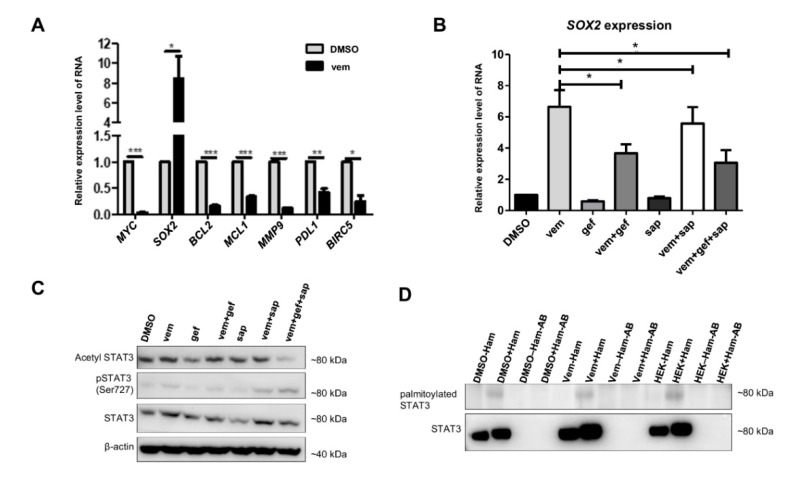
The expression of STAT3 target genes as well as STAT3 modifications are influenced by vem. (**A**) Relative expression levels of STAT3 target genes upon DMSO or vem (3 µM) (24 h) treatment measured by qPCR. (**B**) Inhibition of HER family members can also reduce the expression of *SOX2*, a downstream target of STAT3, in A375 cells as shown by qPCR (relative expression level of RNA, normalized to 18S). (**C**) A375 cells were treated for 24 h with DMSO, vem (3 µM), gef (10 µM), and sap (10 µM). STAT3 Ser727 phosphorylation and Lys685 acetylation are only slightly increased upon vem treatment. Inhibitors of HER family members only slightly influence the phosphorylation status. The same samples are analyzed in Figure 1E for HER3 and STAT3Tyr705 phosphorylation. (**D**) STAT3 palmitoylation was determined after 24 h of DMSO or vem (3 µM) treatment of A375 cells. HEK cells served as positive control. −HAM (hydroxylamine) and −AB (antibody) are negative controls. No difference in STAT3 palmitoylation was observed (*p* < 0.05 *; *p* < 0.01 **; *p* < 0.001 ***).

Analyzing the expression of known STAT3 target genes in different melanoma cell lines upon vem treatment, we found a significant reduction of the expression levels of most genes; in contrast, *SOX2* expression was significantly upregulated (Figure 4A, Supplementary Figure S4). Moreover, the vem-induced *SOX2* expression was diminished in the presence of the HER inhibitors gef or sap, or a combination of both (Figure 4B). These findings demonstrated that the vem-induced expression of the STAT3 target gene *SOX2* is dependent on HER family activation.

Interestingly, vem induced only slight STAT3 phosphorylation at the second phosphorylation site, Ser727 (Figure 4C). Furthermore, STAT3 acetylation at Lys685 was only marginally increased upon vem treatment (Figure 4C). The latter STAT3 modifications were only slightly altered by vem treatment or in the presence of HER3 inhibitors.

In addition to phosphorylation and acetylation, STAT3 can also be palmitoylated, which facilitates the transfer of STAT3 from the cytoplasm to the nucleus [12]. We observed that vem treatment had no influence on STAT3 palmitoylation (Figure 4D).

Thus, the major vem-dependent STAT3 modification was phosphorylation at Tyr705, which caused an increased expression of *SOX2*, whereas other known STAT3 target genes such as *BCL2* were significantly downregulated.

## 3. Discussion

In the present work, we showed that (1) vem induced the activation of HER3 and thereby the upregulation of pSTAT3 and its target gene *SOX2*, (2) HER family inhibition in addition to vem treatment causes increased death of vem-resistant cells, (3) HER3 was activated by a ligand larger than 3 kDa that was cleaved by sheddases, and (4) the major STAT3 modification influenced by vem treatment is phosphorylation at Tyr705. These mechanistic data help to understand how melanoma cells evade targeted therapy and suggest that the inhibition of HER family and especially HER3 could prevent resistance toward targeted therapies by overcoming adaptive resistance.

It was previously demonstrated that HER3 was upregulated following BRAF and MEK inhibitor treatment [17,18,22]. Miller et al. [15] observed reduced shedding of RTKs and HER family members as a posttranslational mechanism of resistance to kinase inhibitor treatment, leading to an augmented surface expression of these receptors. In our experiments, we also revealed an increase of HER3 at the cell surface. Moreover, Reschke et al. [23] found that HER3 expression is increased in metastatic melanoma compared to that in primary melanoma and that HER3 is associated with poor prognosis suggesting the importance of HER3 in melanoma progression. However, the biological significance of the increase of HER3 expression on the cell surface remains to be determined.

In another study, it was found that STAT3 and HER3 activation are interconnected in lung adenocarcinoma [24]. This observation is in agreement with our data on the activation of HER3 and STAT3 with the BRAF inhibitor vem. In addition, in a previous work, we identified STAT3 activation followed by the induction of *SOX2* and *CD24* expression as a mechanism to escape BRAF inhibitor treatment [8]. We found that the blockade of STAT3 activation made melanoma cells more sensitive toward the inhibitor treatment. In the present work, we were able to extend these findings by showing that the inhibition of the HER family also diminished STAT3 activation and suppressed the expression of the STAT3 target gene *SOX2*. At the same time, the sensitivity toward vem treatment was increased in parental as well as in resistant melanoma cells. Our findings imply that the blockage of the HER family upstream of STAT3 could prevent adaptive resistance toward vem treatment. Similar observations were reported by Abel et al. [17], who suggested HER3 activation as a mechanism of adaptive resistance.

To investigate how HER3 was activated in response to vem, we screened different known ligands of HER3. Using size fractionation of vem CM, we found that the protein causing HER3 and STAT3 activation had the molecular weight larger than 3 kDa and was sensitive to heat treatment (Figure 3C). This suggested that the effect was caused most likely by a protein or a protein fragment.

Fattore et al. (2013) [18] showed that the upregulation of phosphorylated HER3 was due to an increased expression of *NRG* by melanoma cells. Furthermore, NRG1 was shown to drive resistance to MEK inhibitors in metastatic uveal melanoma [25] making it a likely candidate. Although the increase in phosphorylation of HER3 is in agreement with our data, we did not observe an increased expression of *NRG* at the mRNA level in any of the tested melanoma cell lines. We also noted that treatment with recombinant NRG1 did not promote HER3 signaling in the melanoma cell lines utilized in our experiments. In contrast, Capparelli et al. [19] found that NRG1 triggered HER3 signaling in mutant BRAF melanoma. However, it should be noted that the NRG1 used in that work was derived from fibroblasts, which was not the case in our study. Moreover, another study showed that fibroblast-derived NRG1 caused anti-androgen resistance in prostate cancer cells. This could be circumvented by pharmacological blockade of the NRG1–HER3 axis [26]. Additionally, it was found in ovarian cancer that NRG1 activated HER3 and promoted proliferation and cancer progression. Inhibition of HER3 could significantly inhibit tumor growth [27]. Thus, this shows the importance of HER3 in tumor progression and therapy resistance.

Most ligands need to be shed from the cell surface by sheddases, which in turn activate important signaling cascades [20]. Since TACE/ADAM17 and ADAM10 were shown previously to play an important role in ligand and receptor shedding [28], we analyzed A375 and SK Mel 28 melanoma cells and found that they expressed ADAM10 but not TACE/ADAM17. Moreover, by blocking ADAM10 by sheddase inhibitors we found that the vem-induced activation of HER3 and STAT3 was diminished, confirming the role of shed ligands in HER3 activation. This finding is also in agreement with the study from Kirkegaard et al. [29] demonstrating that the Matrix Metallopeptidase(MMP) inhibitor BB94 blocks the activation of HER3 and ERK and, thereby, tumor cell growth in breast cancer.

Since most STAT3 target genes except *SOX2* are downregulated upon vem treatment, we analyzed different modifications of STAT3 beyond the canonical STAT3 activation by phosphorylation at Tyr705. The alternative STAT3 modifications (non-canonical STAT3 activation) are acetylation at Lys685 or phosphorylation at Ser727, which were reported to play an important role in cancer progression [10,11,30]. In our study, we showed that none of these modifications played a major role in vem-induced STAT3 activation. Since Niu et al. [12] pointed out the importance of STAT3 palmitoylation for the translocation to the nucleus, we analyzed the palmitoylation upon vem treatment in more detail. However, no differences in STAT3 palmitoylation between vem-treated and control cells were detected (Figure 4D). Thus, STAT3 phosphorylation at Tyr705 seems to be mainly involved in vem-induced STAT3 activation.

We conclude that HER3 activation and thereby STAT3 activation play an important role in adaptive resistance, and inhibition of HER family members could help to overcome resistance against targeted therapies and, thereby, extend the duration of a successful treatment of patients.

## 4. Materials and Methods

### 4.1. Cell Culture

All human melanoma cell lines used (A375, SK Mel 28, and HT144) were BRAF^V600E^-mutated and obtained from ATCC, Manassas, VA, USA. The cells were cultured in DMEM (Gibco, Life Technologies, Carlsbad, CA, USA) supplemented with 10% heat-inactivated Fetal Bovine Serum (FBS) (Biochrom, Cambridge, UK), 0.1 mM β-mercaptoethanol (Gibco, Life Technologies), 1% non-essential amino acids (NEAA) and 1% penicillin/streptomycin (Sigma-Aldrich, St. Louis, MO, USA). A375 cells resistant to vem (A375-res) were cultured in the same culture medium with the addition of 7 µM vem. The cell lines were cultured in a humidified incubator at 37 °C and 5% CO_2_. The cells were sub-cultured when reaching a confluency of roughly 80% (approximately every 3–5 days).

### 4.2. Cell Viability Assay

The cells were seeded at a density of 2500 cells/well (72 h) or 5000 cells/well (24 h) in flat bottom 96-well plates (Gibco, Life Technologies). The next day, the cells were treated with increasing concentrations of vem (1 nM up to 10,000 nM). To compare the sensitivity to vem alone with the sensitivity to a combination of vem and gefitinib (EGFR inhibitor, which by inhibiting EGFR influences dimerization of EGFR with HER2/3/4 dissolved in DMSO, Selleckchem, Houston, TX, USA), vem and sapitinib (mainly Her2/3 inhibitor dissolved in DMSO; Selleckchem), or a combination of vem, gefitinib, and sapitinib, 10 µM of gefitinib and/or sapitinib were added to the different concentrations of vem. After 24 or 72 h of treatment, alamarBlue (10% of the total volume) was added to each well. The cells were incubated for 4 h at 37 °C. A SpectraMax M5 microplate reader (Tecan, Männedorf, Switzerland) was used to measure the fluorescence at an excitation wavelength of 535 nm and an emission wavelength of 590 nm. The measured fluorescence intensity was normalized to the DMSO control (100%) to calculate the viability. Prism was used to calculate the IC_50_ and plot the concentration vs. the viability for all conditions.

To calculate whether the effects are synergistic or additive, 5000 cells/well of A375 and A375-res cells were seeded in flat bottom 96-well plates. The next day, the cells were treated with increasing concentrations of vem, gefitinib, and sapitinib, alone or in combination, in increasing concentrations ranging from 1 nM up to 20,000 nM as indicated in the figures. After 24 h of treatment the cell viability was analyzed as described above.

### 4.3. Proteome Profiler Human Phospho-RTK Array Kit

Briefly, 1 × 10^6^ cells were seeded in a 10 cm dish. Upon attaching to the surface, they were treated for 24 h with vem (3 μM) or DMSO as a control. Next (24 h later), the cells were lysed, and the array was performed according to the manufacturer’s instructions (R&D systems, Minneapolis, MN, USA; Cat # ARY001B).

### 4.4. Inhibitor Treatment

For this, 1 × 10^6^ cells were seeded in a 10 cm dish and treated for 24 h with DMSO, 3 µM vem, 10 µM sapitinib, or 10 µM gefitinib or with combinations of these inhibitors as indicated.

For the treatment with DMSO, vem (3 µM), mibefradil (7 µM) (T-type Ca Channel inhibitor), GI254023x (5 µM) (ADAM10 inhibitor), and BB94 (10 µM) (MMP inhibitor) 1 × 10^6^ cells were seeded in a 10 cm dish and then treated for 24 h.

### 4.5. Pulse Experiment

Briefly, 1 × 10^6^ cells were seeded in a 10 cm dish. The next day, the cells were treated for 6 h with vem (3 µM) or DMSO as a control. The cells were washed twice with pre-warmed (37 °C) medium before fresh medium was added. After overnight incubation, the supernatant was filtrated (0.22 µm). To remove extracellular vesicles (EVs) from half of the supernatant, ultracentrifugation (2 h at 100,000× *g*) was used. Next, the supernatant was filtered with Amicon filters with a cutoff of 3 kDa to ensure that no vem was transferred. Material not passing the filter due to a molecular weight larger than 3 kDa was resolved in fresh medium and transferred to naive cells seeded the day before at a density of 2 × 10^5^ cells/well in a 6-well plate. The cells were treated with the supernatants for 24 h and were then harvested to perform Western blot analysis.

### 4.6. Lipofectamine Transfection

At the time of transfection, cells were around 80% confluent. For transfection, Lipofectamine™ RNAiMAX Transfection Reagent (Thermo Fisher Scientific, Waltham, MA, USA) was used following the manufacturer’s protocol. The following siRNAs obtained from Eurofins Genomics GmbH were used (Table 1): 

To monitor transduction efficiency qPCR and Western blot analyses were utilized.

### 4.7. Palmitoylation Assay

Protein palmitoylation was analyzed using an assay described in detail elsewhere [31]. In brief, cells were seeded in 15 cm dishes and were treated for 24 h with DMSO or vem (3 µM) after reaching 80% confluency. The next day, cells were trypsinized, and the pellet was washed once with Phosphate-buffered saline (PBS) before lysing for 30 min in Radioimmunoprecipitation assay buffer (RIPA) buffer containing a protease inhibitor cocktail (Roche) as well as 50 mM *N*-ethylamine (Sigma). After 30 min of centrifugation at 16,000× *g*, the supernatant was harvested. The protein concentration was measured by Bicinchoninic acid assay (BCA) and 1000 µg per sample were used for immunoprecipitation overnight at 4 °C utilizing a mouse monoclonal STAT3 antibody (Cell Signaling; Cat # 9139S) (final dilution 1:200). As a control, 1000 µg protein of each sample were incubated overnight without the STAT3 antibody (−AB control). The next day, the antibody-bound STAT3 protein as well as the −AB controls were precipitated with Dynabeads (Invitrogen, Carlsbad, CA, USA). Next, hydroxylamine (HAM) cleavage and acyl-biotin exchange was performed as described [31]. In control samples, the treatment with HAM was omitted (−HAM). To analyze the results, SDS-Page and Western blotting using streptavidin–peroxidase and STAT3 immunolabeling were performed. 

### 4.8. Statistical Analysis

All statistical analyses were performed on at least three independent experiments using GraphPad Prism (GraphPad Software, San Diego, CA, USA). Data are expressed as means ± SEM. Differences were either analyzed by *t*-test, if two conditions were compared, or by ANOVA with post hoc Bonferroni test if more than two conditions were compared. Synergism analysis was performed based on Loewe additivity and the associated combination index (CI) with 95% confidence interval. Drugs are considered to act synergistically if the upper confidence limit of the CI is below 1. The combination index was estimated according to Chou and Talalay (1984) [32] using the implementation by Lee et al. (2007) [33]: https://biostatistics.mdanderson.org/SoftwareDownload/SingleSoftware/Index/18.

### 4.9. Additional Methods

These additional methods are described in the Supplementary Materials: RNA isolation, cDNA synthesis, qPCR, protein isolation, Western blotting, flow cytometry.

## 5. Conclusions

Taken together, STAT3 activation via HER3 seems to be a mechanism that melanoma cells adapt to escape from targeted therapies. Since it is easier to target an RTK at the cell surface (HER) than a transcription factor (STAT3), the inhibition of HER family and specifically HER3 resulting in less STAT3 activation might help to circumvent resistance to BRAF inhibitor treatment.

## Figures and Tables

**Table 1:** siRNAs used in this study.

**siGFP**	5′-GGCCAGGUCCAGCAGCGCACCUU-3′
**siHER3.1**	5′-GTGAGGTGGTGATGGGGAA-3′
**siHER3.2**	5′-CCATCTTCGTCATGTT GAA-3′

## Supplementary Materials

The following are available online at https://www.mdpi.com/2072-6694/12/12/3761/s1, Supplementary Figure S1: HER3 and STAT3 are activated upon vem treatment; Supplementary Figure S2: Inhibition of HER family members leads to increased sensitivity toward vem treatment; Supplementary Figure S3: Expression of HER family members and their ligands. S4: All uncropped western blots. These additional methods are described in the supplement: RNA isolation, cDNA synthesis, qPCR, protein isolation, Western blotting, flow cytometry.
