# Supplementary material for: HER3-Receptor-Mediated STAT3 Activation Plays a Central Role in Adaptive Resistance toward Vemurafenib in Melanoma
Source: Cancers (Basel). 2020 Dec 14;12(12):3761. doi: 10.3390/cancers12123761 (PMC7764938; doi:10.3390/cancers12123761)

# Figure 1B

After transfere the blot was cut in different pieces according to size

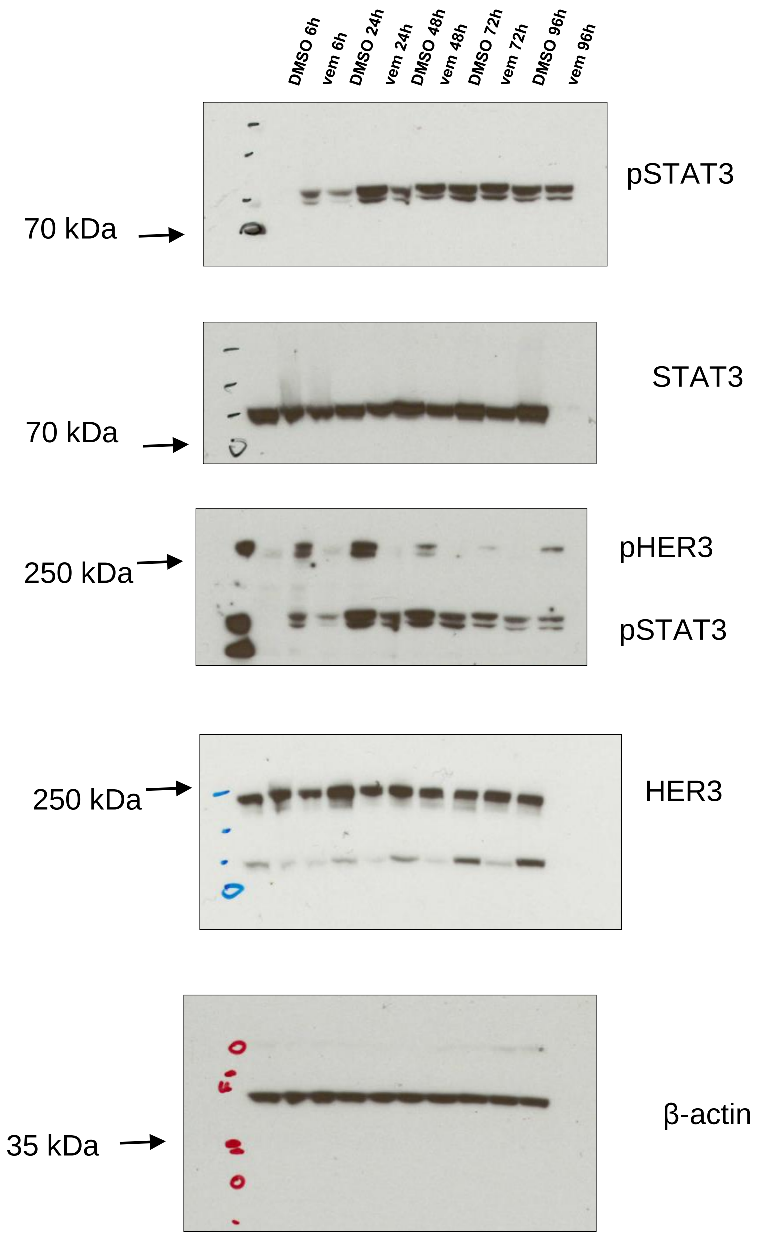

# Figure 1D

After transfere the blot was cut in different pieces according to size

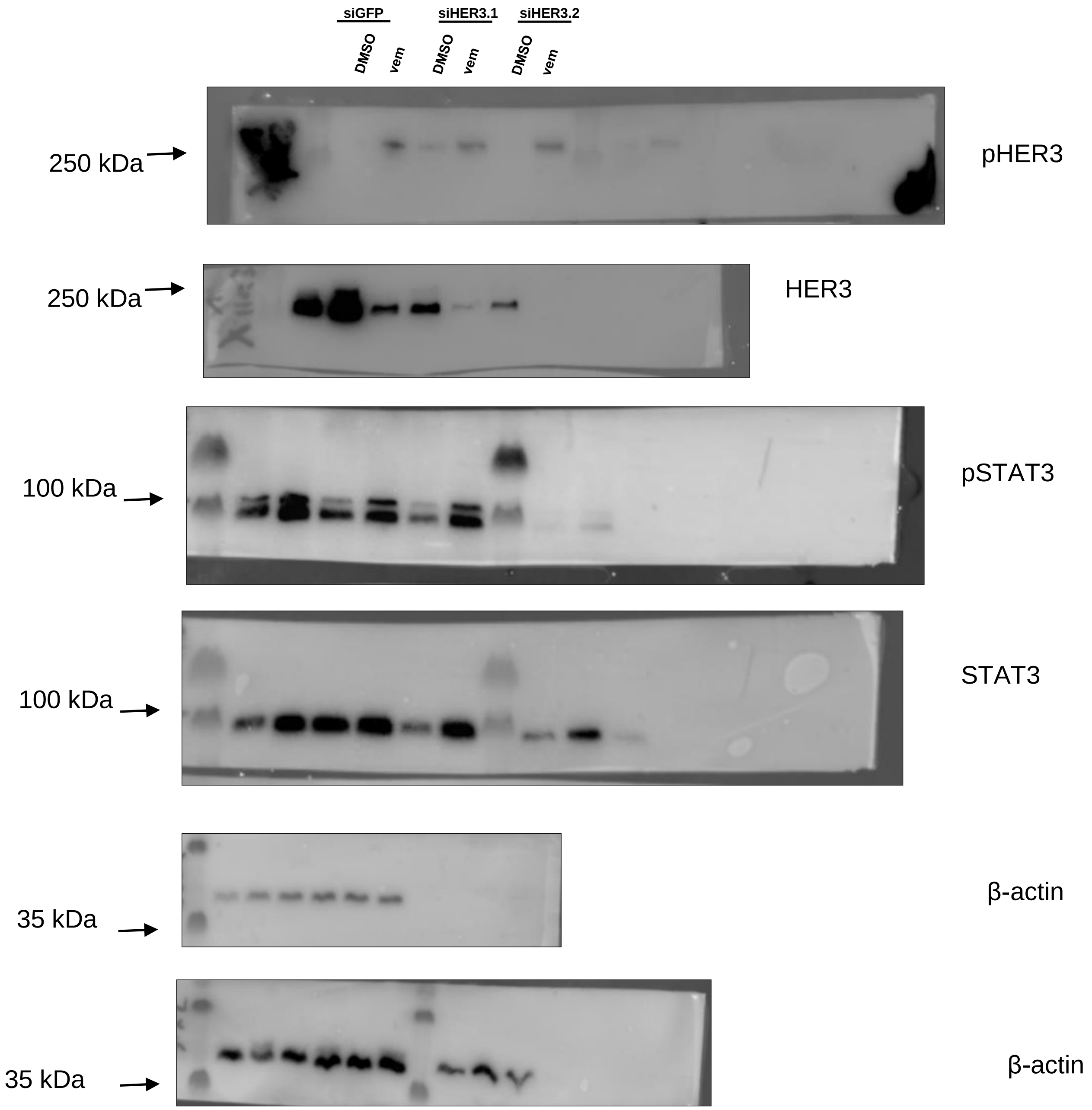

# Figure 1E

After transfere the blot was cut in different pieces according to size

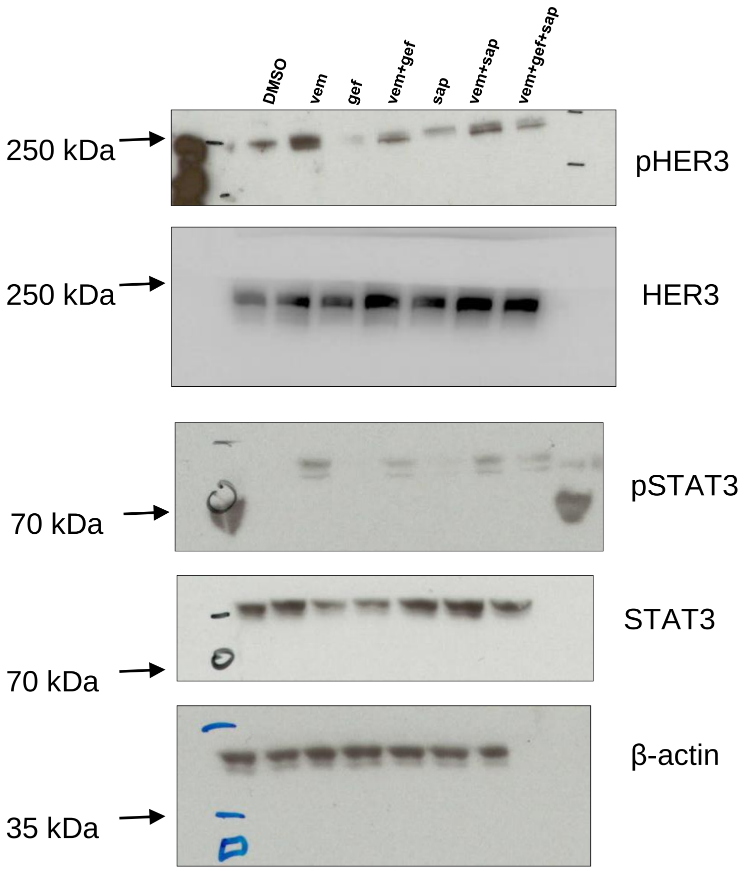

# Figure 3C left

After transfere the blot was cut in different pieces according to size

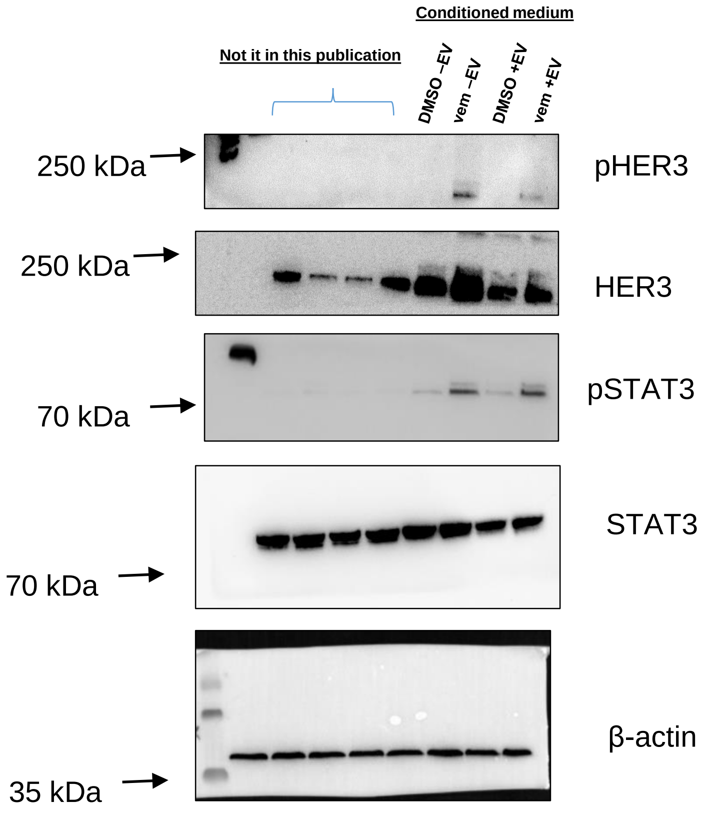

# Figure 3C right

After transfere the blot was cut in different pieces according to size

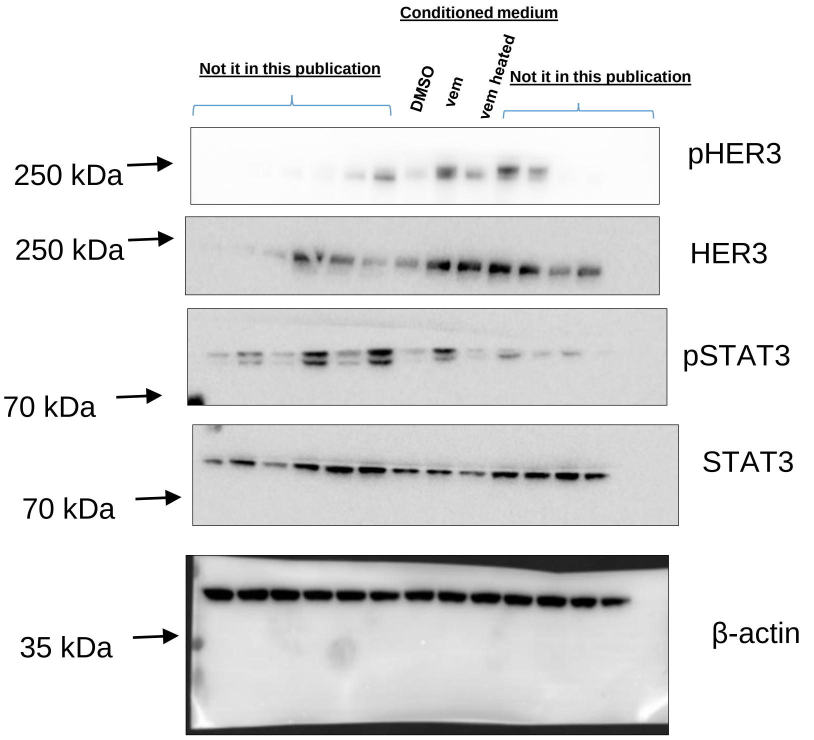

# Figure 3E

After transfere the blot was cut in different pieces according to size

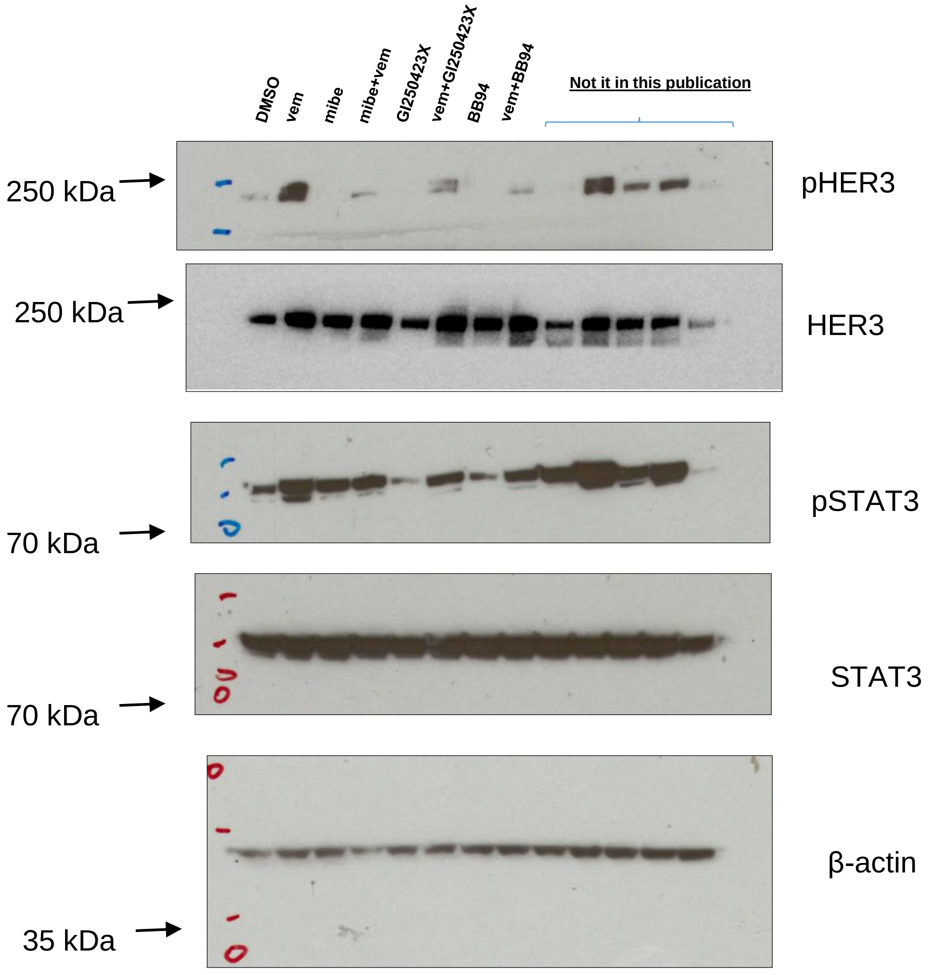

**Figure 4C**

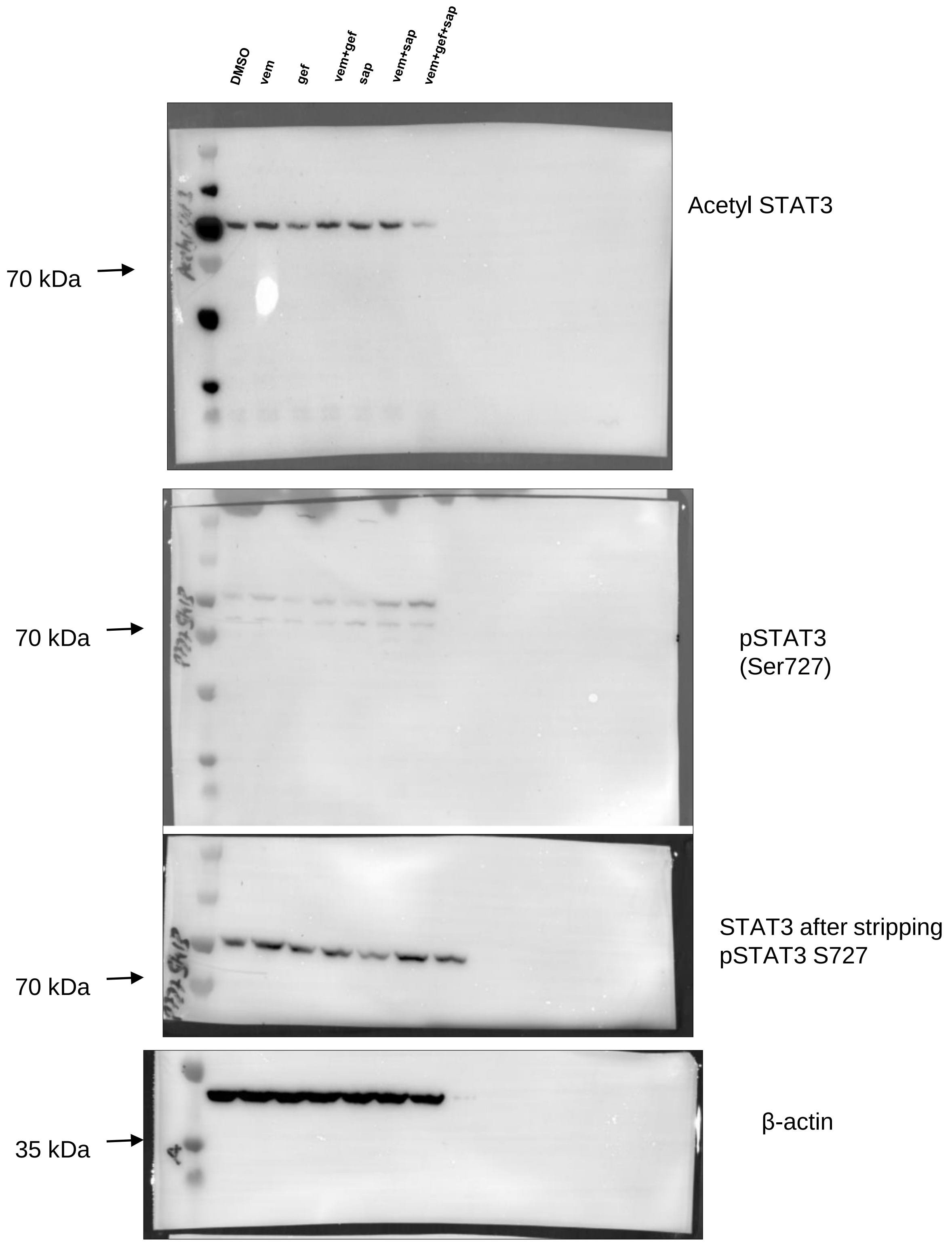
DMSO
vem
gef
vem+gef
sap
vem+sap
vem+gef+sap
70 kDa
Acetyl STAT3
70 kDa
pSTAT3
(Ser727)
70 kDa
STAT3 after stripping
pSTAT3 S727
35 kDa
β-actin

**Figure 4D**

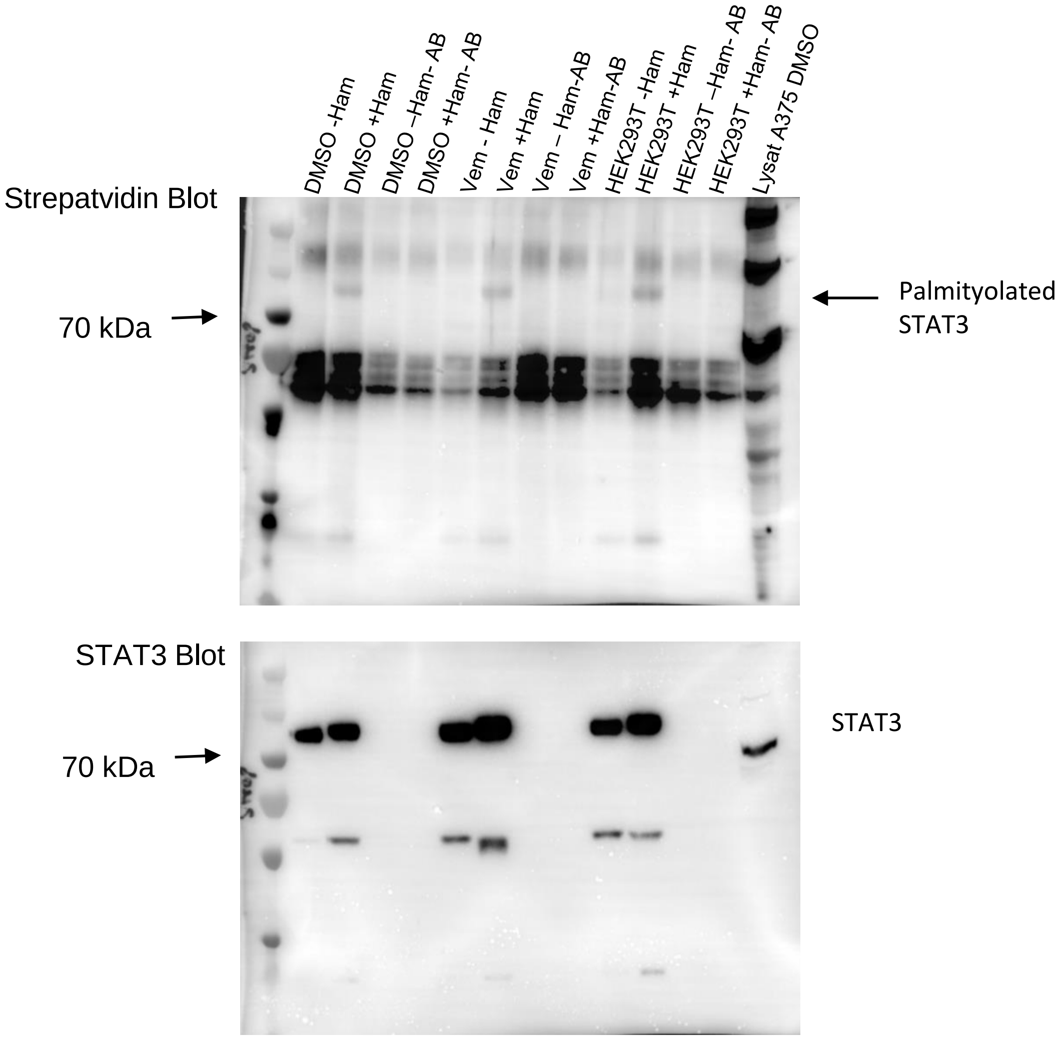

DMSO -Ham
DMSO +Ham
DMSO –Ham- AB
DMSO +Ham- AB
Vem - Ham
Vem +Ham
Vem – Ham-AB
Vem +Ham-AB
HEK293T -Ham
HEK293T +Ham
HEK293T –Ham- AB
HEK293T +Ham- AB
Lysat A375 DMSO
Strepatvidin Blot
70 kDa
Palmityolated STAT3
STAT3 Blot
70 kDa
STAT3

# Suppl. Fig 1B

After transfere the blot was cut in different pieces according to size

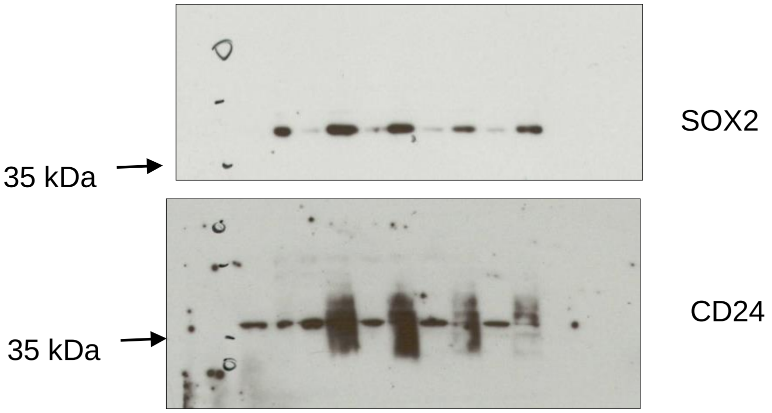

# Suppl. Fig1C

After transfere the blot was cut in different pieces according to size

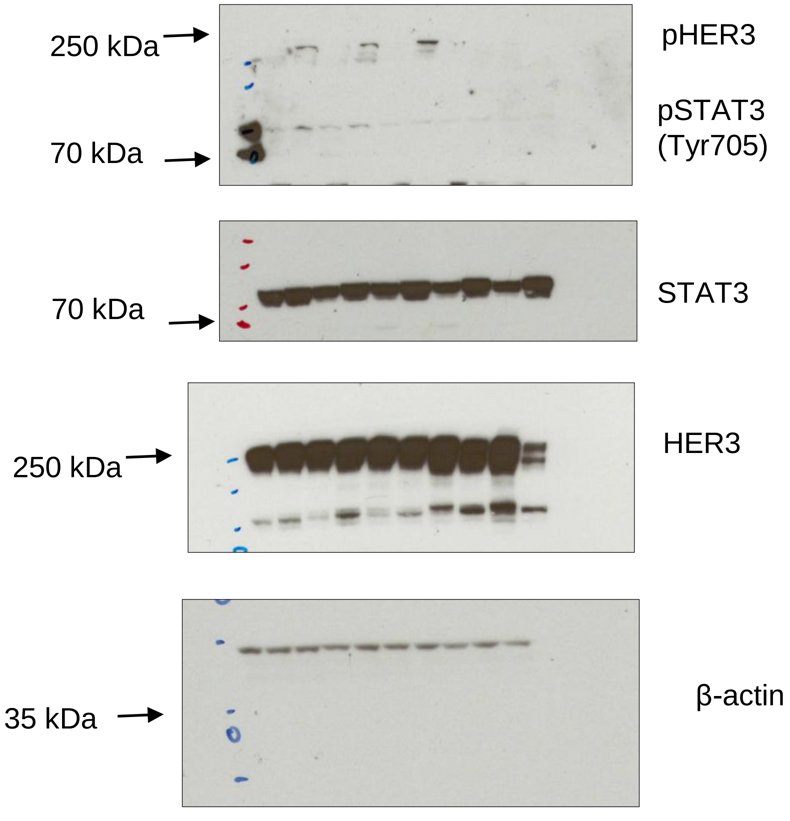

**Suppl. Fig 1D**

After transfere the blot was cut in different pieces according to size

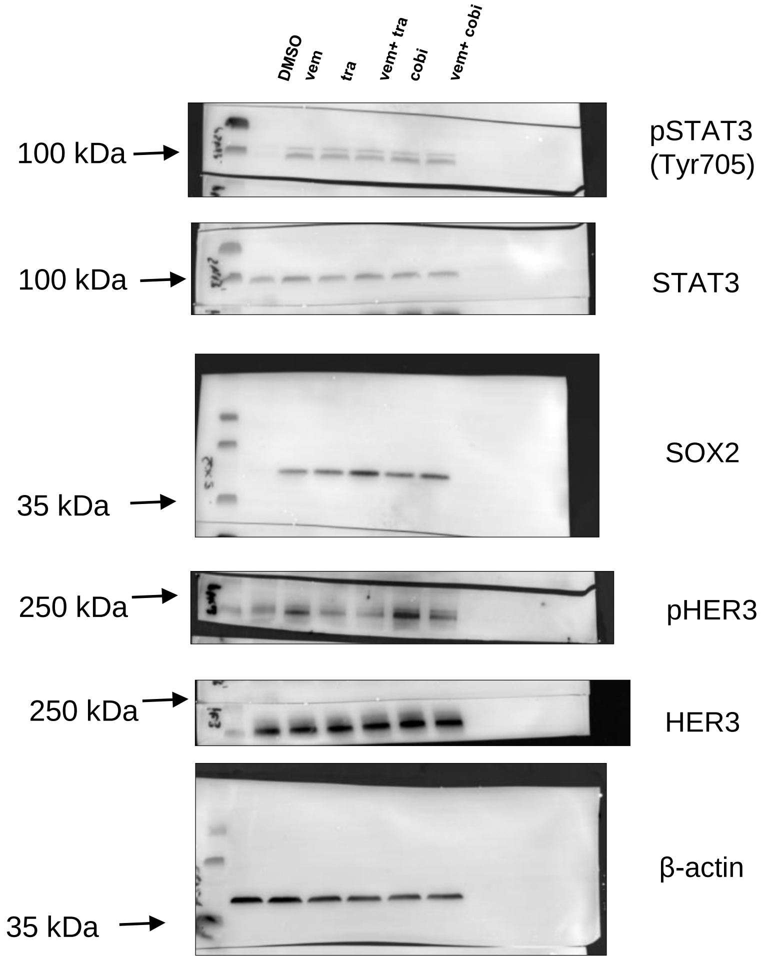

# Blots additionally to 1E quantified for Fig. 1F

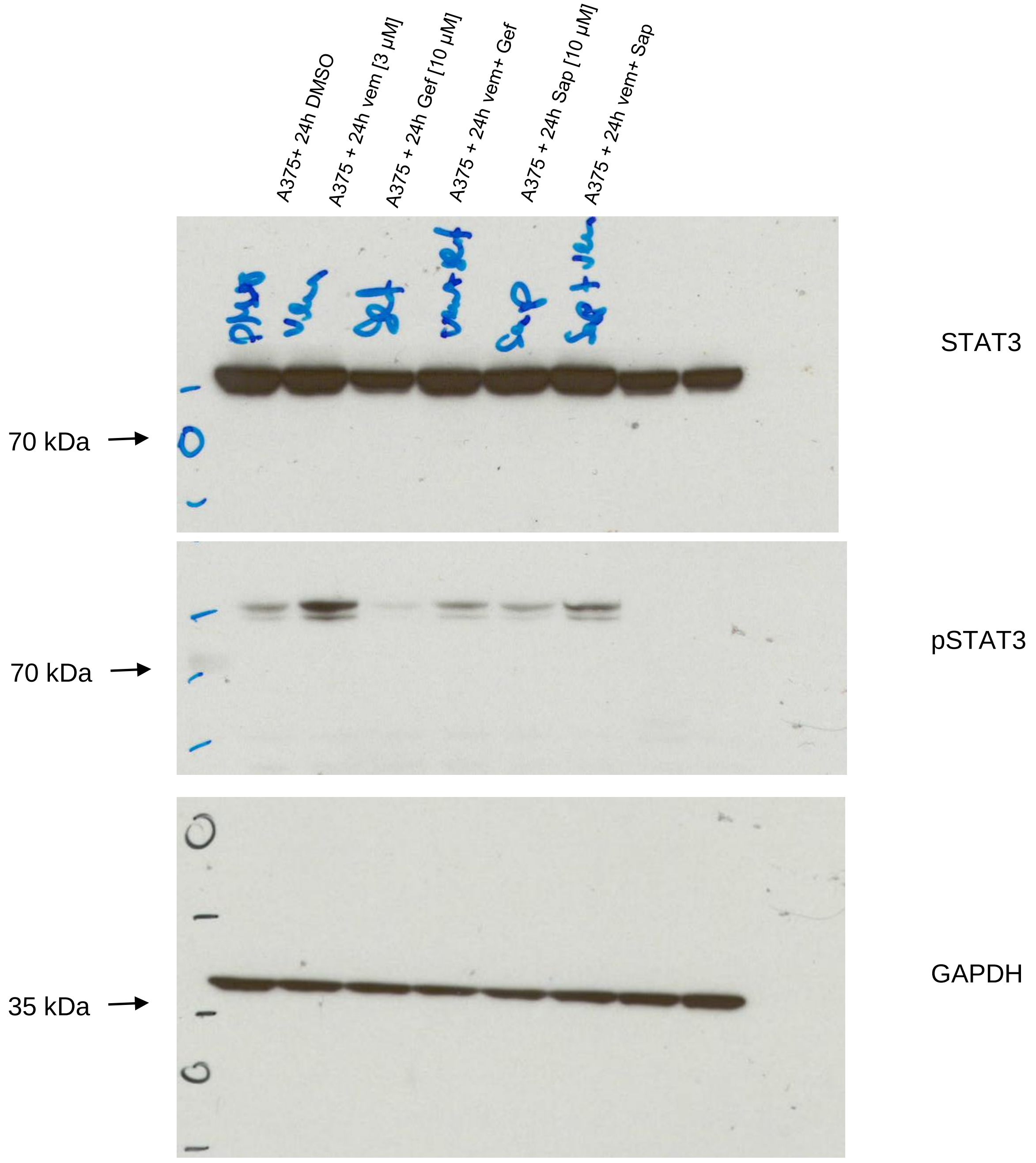

# Blots additionally quantified for Fig. 1F

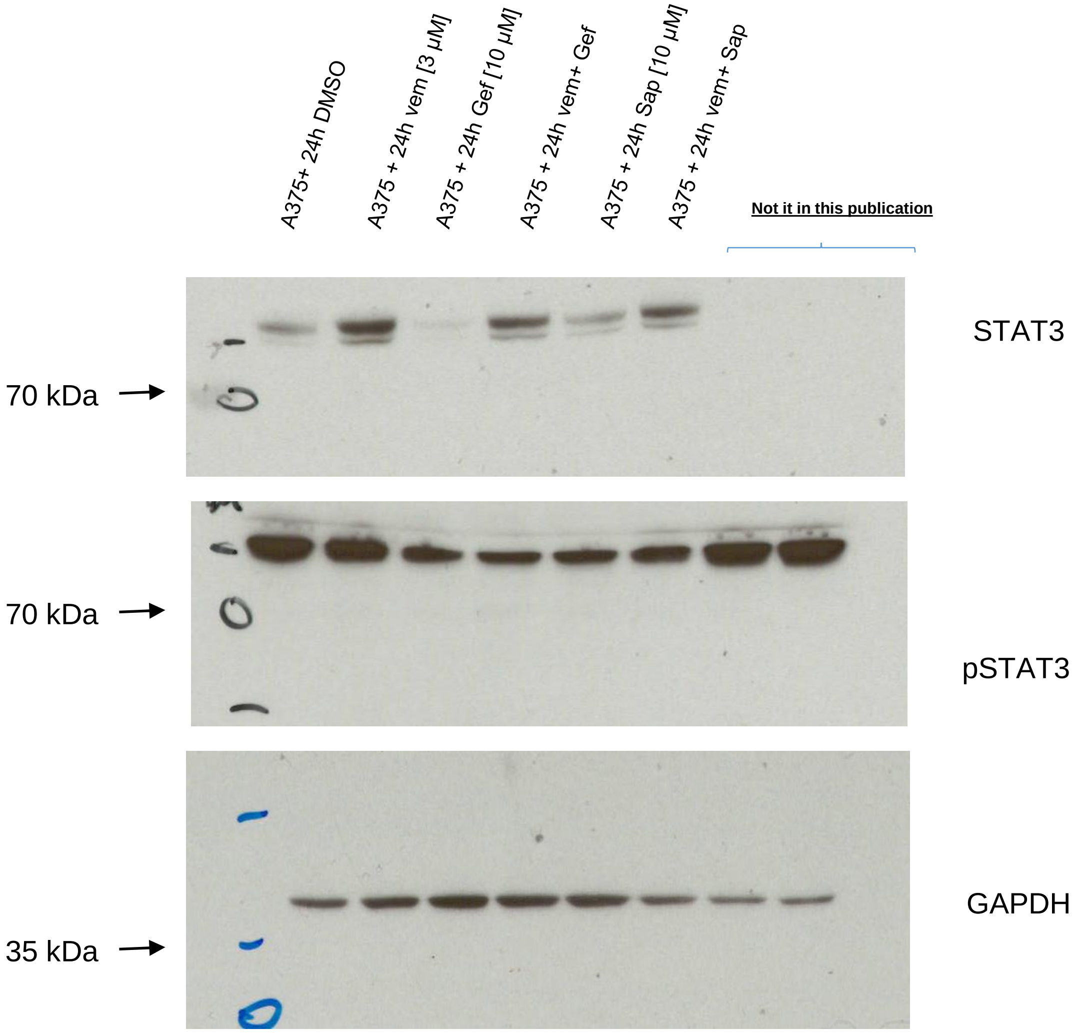

## Blots additionally quantified for Fig. 1F

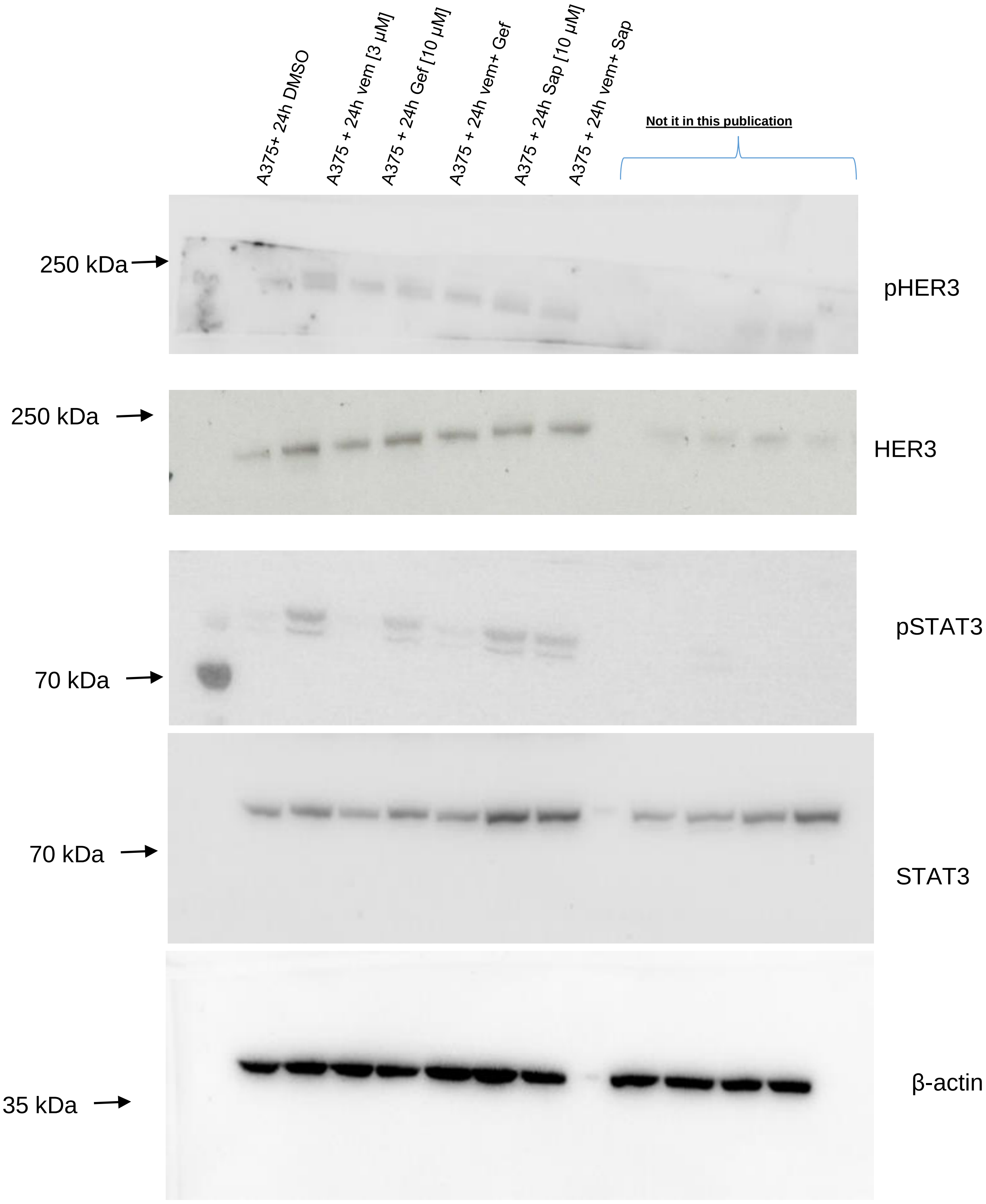

# Blots additionally quantified for Fig. 1F

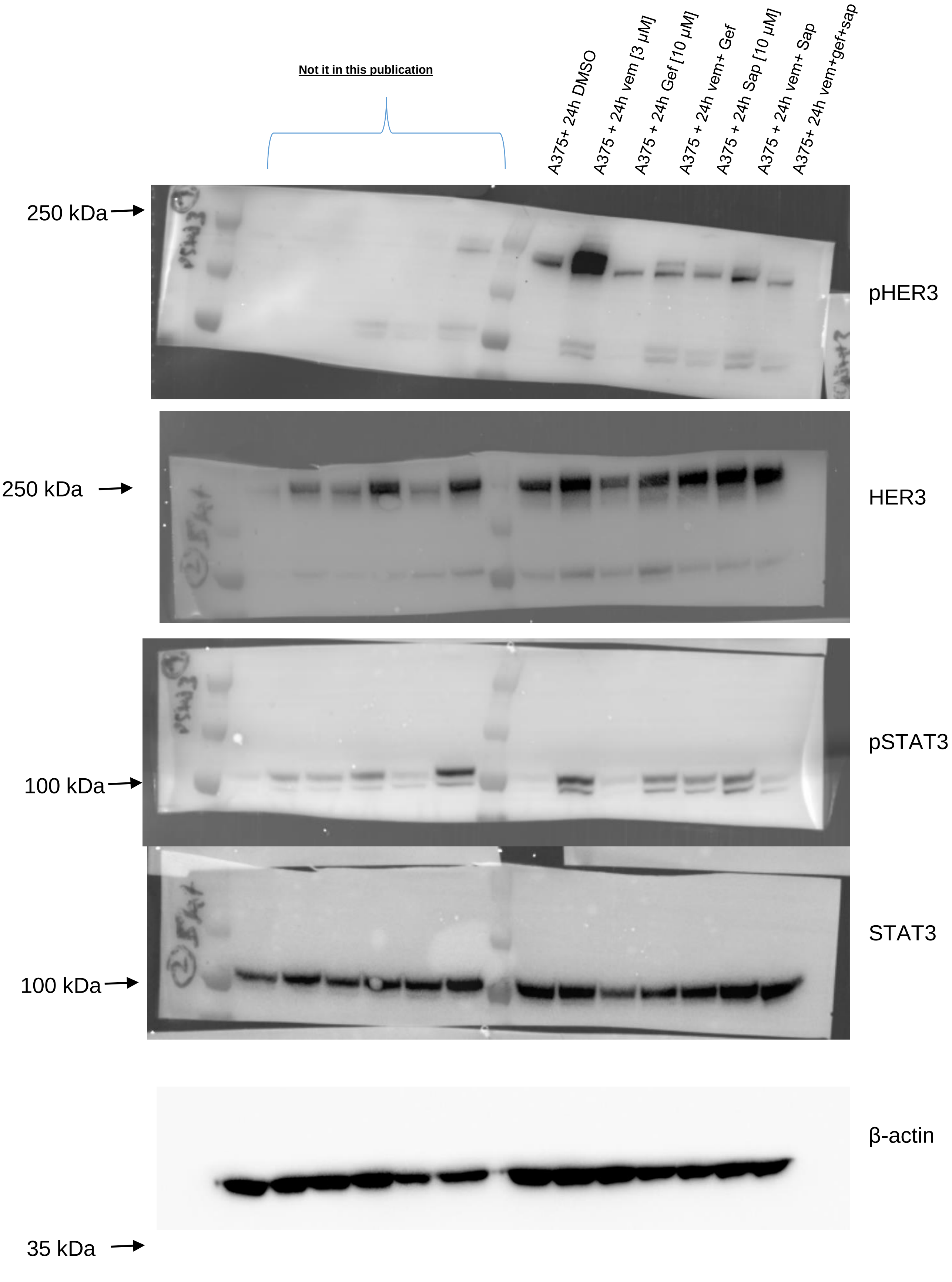

Supplement: Supplementary file 1 [file cancers-12-03761-s001.zip › cancers-974568_supplementary-S4.pdf]
