# Supplementary material for: HER3-Receptor-Mediated STAT3 Activation Plays a Central Role in Adaptive Resistance toward Vemurafenib in Melanoma
Source: Cancers (Basel). 2020 Dec 14;12(12):3761. doi: 10.3390/cancers12123761 (PMC7764938; doi:10.3390/cancers12123761)
Supplement: Supplementary file 1 [file cancers-12-03761-s001.zip › cancers-974568_supplementary.pdf]

cancers

Supplemental Methods

RNA isolation and cDNA synthesis

The RNeasy Mini Kit (Qiagen, Hilden Germany) was used to isolate total RNA following the manufacturer's instructions including DNase digestion on column. The concentration of the isolated RNA was measured with the NanoDrop ND1000 spectrophotometer (PEQLAB Biotechnologie GmbH, Erlangen, Germany). 500 ng RNA were used as a template for cDNA synthesis with the Revert Aid First Strand cDNA synthesis kit (Thermo Fisher Scientific, Waltham USA) following the manufacturer's protocol.

qPCR

Quantitative real-time PCR (qPCR) was used to determine the expression level of mRNA. cDNA was mixed with the SYBR Green Master Mix (Applied Biosystems, Life technologies, Carlsbad USA) and the primer pair of interest and the PCR was performed with a 7500 real-time PCR system (Applied Biosystems, Life Technologies). The housekeeping gene 18S was used as an endogenous control in all experiments. To obtain the relative expression levels all CT values were normalized to 18S before the $2^{-\Delta\Delta Ct}$ was calculated. The mean of technical replicates of at least 3 independent experiments was used to calculate the mean and the SEM as well as statistical significance.
The following primer pairs were used:

SOX2 forw	GCCGAGTGGAAACTTTTGTCG
SOX2 rev	GGCAGCGTGTACTTATCCTTCT
18S forw	GAGGATGAGGTGGAACGTGT
18S rev	TCTTCAGTCGCTCCAGGTCT
BCL2 forw	GCTACCGTCGTGACTTCGC
BCL2 rev	CCCCACCGAACTCAAAGAAGG
BCL2L1 forw	GGTCGCATTGTGGCCTTTTTC
BCL2L1 rev	TGCTGCATTGTTCCCATAGAG
MMP9 forw	GGGACGCAGACATCGTCATC
MMP9 rev	TCGTCATCGTCGAAATGGGC
MCL-1 forw	AACAAAGAGGCTGGGATGGGTTTG
MCL-1 rev	AAACCAGCTCCTACTCCAGCAACA

BIRC5 forw	AGGACCACCGCATCTCTACAT
BIRC5 rev	AAGTCTGGCTCGTTCTCAGTG
NRG1 forw	TGTTAAGAAACTCGCCAATAGCC
NRG1 rev	GCTGTCCACTTCCAATCTGTTA
EGFR forw	CAG AGTGATGTCTGG AGCTACG
EGFR rev	GGGAGG CGT TCT CCT TTCT
HER2 forw	GCC ATG AGC AGT GTG CTG
HER2 rev	ACA GAT GCC ACT GTG GTT GA
HER3 forw	CAA TCC CCA CAC CAA GTA TCA
HER3 rev	GAT GTT TGA TCC ACC ACA AAG TT
HER4 forw	GCG AGA CAA ACC CAA ACA AG
HER4 rev	CAA TGC TTG AAG GTC TCC ATT
BTC forw	ACT GCA TCA AAG GGA GAT GC
BTC rev	TCT CAC ACC TTG CTC CAA TG
EGF forw	CGC AGG AAA TGG GAA TTC TA
EGF rev	CCA TGA TCA CTG AGA CAC CAG
AREG forw	CGG AGA ATG CAA ATA TAT AGA GCAC
AREG rev	CAC CGA AAT ATT CTT GCT GACA
EREG forw	AGG AGG ATG GAG ATG CTC TG
EREG rev	GAG GAC TGC CTG TAG AAG ATGG
TGFa forw	CCC AGA TTC CCA CAC TCAG-

TGFa rev	ACG TAC CCA GAA TGG CAG AC
NRG2 forw	GGT GGC CTA CTG CAA
NRG2 rev	ACA TGT TCT GCC GGA GGT
MYC forw	CTCCTCCTCGTCGCAGTAGA
MYC rev	GCTGCTTAGACGCTGGATTT

Protein Isolation

The cell pellets were washed once in PBS before resolving in complete mini lysis buffer (Roche, Basel Switzerland) with 1% Triton-X-100 and PhosSTOP (Roche). After 30 min incubation on ice, the samples were centrifuged at 14,000 rpm for 15 min at 4 °C. The supernatant containing the protein was analyzed with the Pierce BCA protein assay kit (Thermo Fisher Scientific) following the manufacturer's instructions to determine protein concentration.

Western Blot

The proteins (30 μg/lane) were separated according to size using SDS-PAGE gel electrophoresis. Next, the proteins were transferred to a PVDF membrane (Merck Millipore, Burlington USA), followed by blocking in 5% milk/TBS-T or 5% BSA/TBS-T depending on the primary antibody for 1h at room temperature. Afterwards, the membrane was incubated overnight at 4 °C with the primary antibody diluted in blocking buffer. The next day, membranes were washed and incubated for 1h at room temperature with HRP-conjugated secondary antibody. Luminata Forte Western HRP substrate (Merck Millipore) was used to visualize the HRP-bound antibody.

The following primary antibodies were used: SOX2 (Cell Signaling) 1:500, STAT3 (Cell Signaling) 1:5000, STAT3 , Lys685 (Cell Signaling) 1:1000, pSTAT3, Tyr705 (Cell Signaling) 1:500, pSTAT3, Ser727 (Cell Signaling) 1:1000, HER3 (Cell Signaling) 1:3000, pHER3 (Cell Signaling) 1:500, CD81 MA5-13548 (Thermo Fisher Scientific) 1:1000, β-actin (Cell Signaling) 1:10,000.

To quantify band intensity ImageJ (Rasband, W.S., ImageJ, U. S. National Institutes of Health, Bethesda, Maryland, USA, https://imagej.nih.gov/ij/, 1997-2018) was used.

Flow Cytometry

One day prior to treatment 2 × 10^5 cells were seeded in 6-well plates. The next day, these cells were treated with 3 μM vem or DMSO as a control for 24h. Next, the cells were trypsinized and transferred to FACS tubes where they were washed with PBS and resolved in FACS buffer (PBS + 0.5% BSA). Followed by incubation for 1h at room temperature with the primary antibody diluted in FACS buffer. After washing, the cells were incubated for 30 min with a PE-conjugated secondary antibody (Jackson ImmunoResearch, West Grove USA). Finally, after washing the cells, they were resuspended in 200 μl FACS buffer and analyzed using FacsCanto II (BD Biosciences, New Jersey USA). The acquired data were analyzed with the FlowJo software. The following antibodies were used: N-terminal HER3 MA5-12867 (Thermo Fisher Scientific) 1:50, TACE ectodomain (R&D systems, Minneapolis USA) 1:20, ADAM10 ectodomain (R&D systems) 2.5 μg/100 μL.

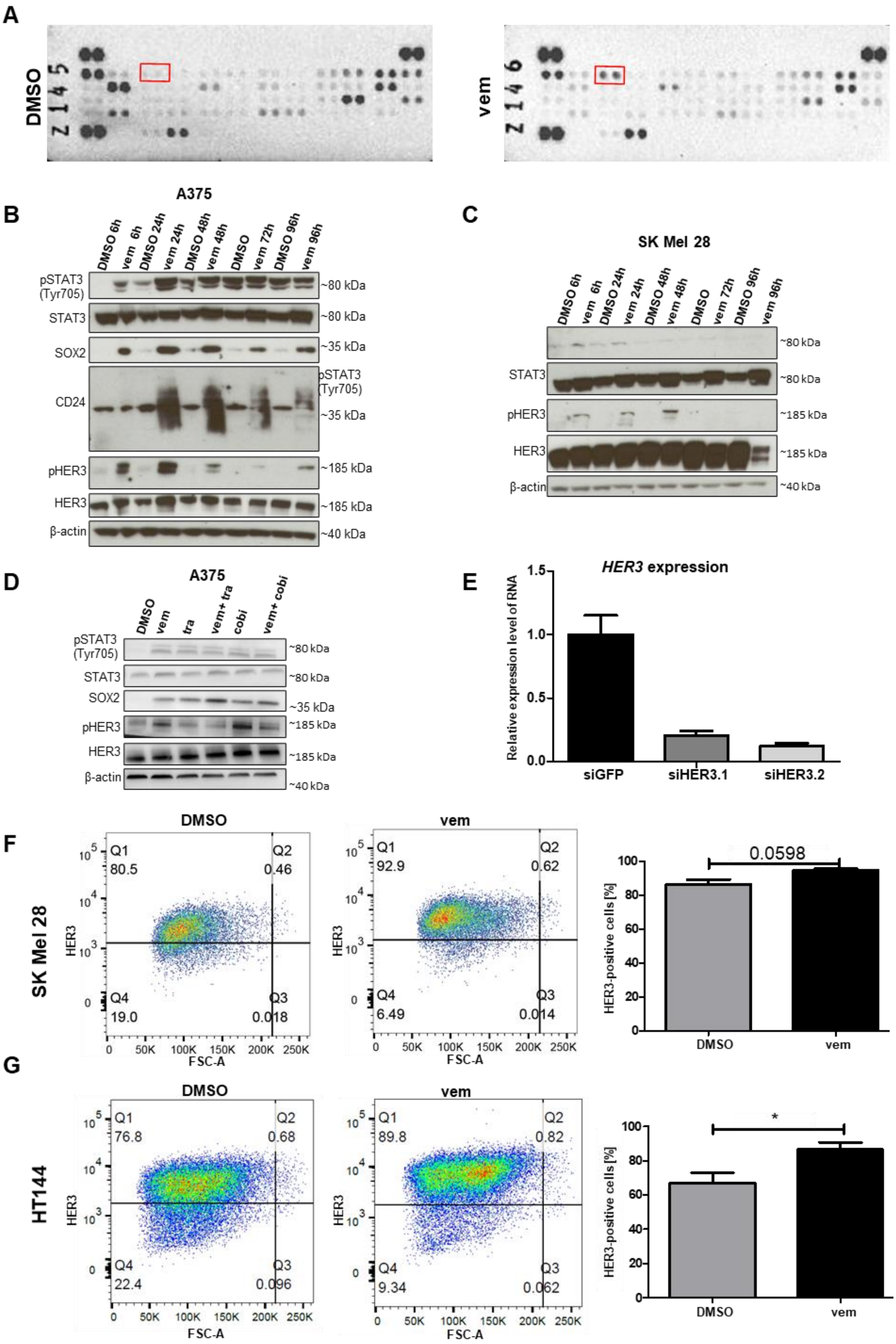

Figure S1: HER3 and STAT3 are activated upon vem treatment. **A:** Proteome Profiler Human Phospho-RTK Array of A375 cells treated for 24h with vem (3 µM) or DMSO as control. pHER3 is marked in red. **B:** A375 cells were treated for 6 to 96h with vem (3 µM) and afterwards the activation of STAT3, HER3 and the expression of the STAT3 downstream targets CD24 and SOX2 was analyzed by western blot (this is the same western blot as shown in Figure 1B for HER3 and STAT3). **C:** SK Mel 28 cells were treated for 6 to 96h with vem [3 µM] to evaluate the STAT3 and HER3 activation upon different times of treatment. **D:** A375 cells were treated for 24 h with 3 µM of vem, trametinib (tra) and cobimetinib (cobi) or in combination. The expression level of (p)HER3, (p)STAT3 and SOX2 was

analyzed by western blot. **E:** Validation of siRNA mediated KD of HER3. Relative expression levels of *HER3* determined by qPCR normalized to 18S. **F/G:** The cell surface expression of HER3 in SK Mel 28 (D) and HT144 (E) cells after 24h of treatment with DMSO or vem [3 μM] was measured by flow cytometry. The experiments revealed an increased expression of HER3 after vem treatment. ($p < 0.05$ *; $p < 0.01$ **; $p < 0.001$ ***).

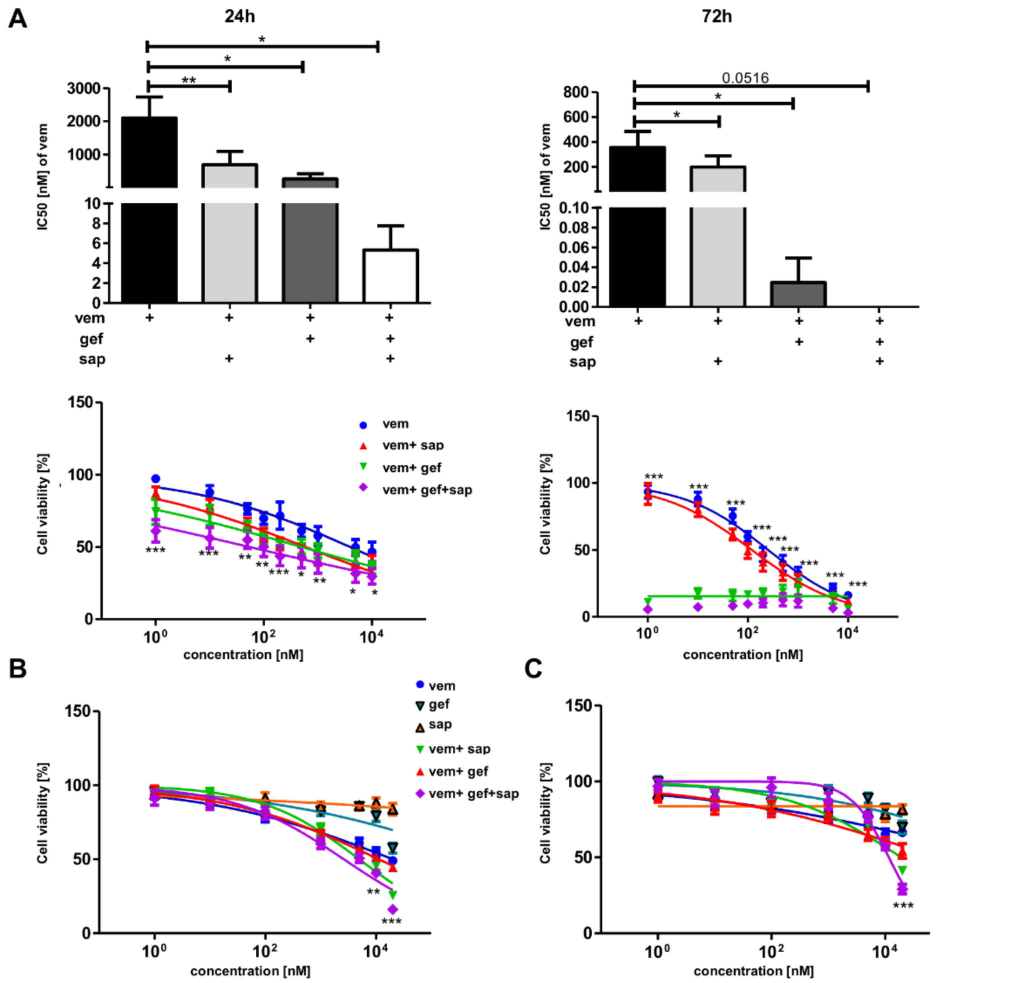

	vem	gef	sap	vem+ gef	vem+ sap	vem+ gef+ sap
IC50	20279	874374	1,152*10[13]	4993	11294	2633

	vem	gef	sap	vem+ gef	vem+ sap	vem+ gef+ sap
IC50	826576	1,91*10[6]	N/A	19256	76324	11572

Figure S2: Inhibition of HER family members leads to increased sensitivity towards vem treatment. **A:** SK Mel 28 cells were treated for 24h with increasing concentrations of vem ± HER inhibitors (gef (10 μM)/sap (10 μM)). Next, the alamarBlue assay was used to determine the does response curves as well as the IC50 values. The IC50 value of each combination was statistically analyzed in comparison to vem alone. In the dose response curves of all biological replicates significance is indicated for the comparison between vem and vem+ gef+ sap. **B/C:** A375 (B) and A375-res (C) cells were treated for 24h with increasing concentrations of vem, gef, sap or combinations of each in the concentration as indicated in equal ratios. Viability was analyzed by alarmaBlue assay and dose response curves and IC50 values of all indicated treatments was determined. Significance is shown for the comparison of vem vs vem+ gef+ sap. ($p < 0.05$ *; $p < 0.01$ **; $p < 0.001$ ***).

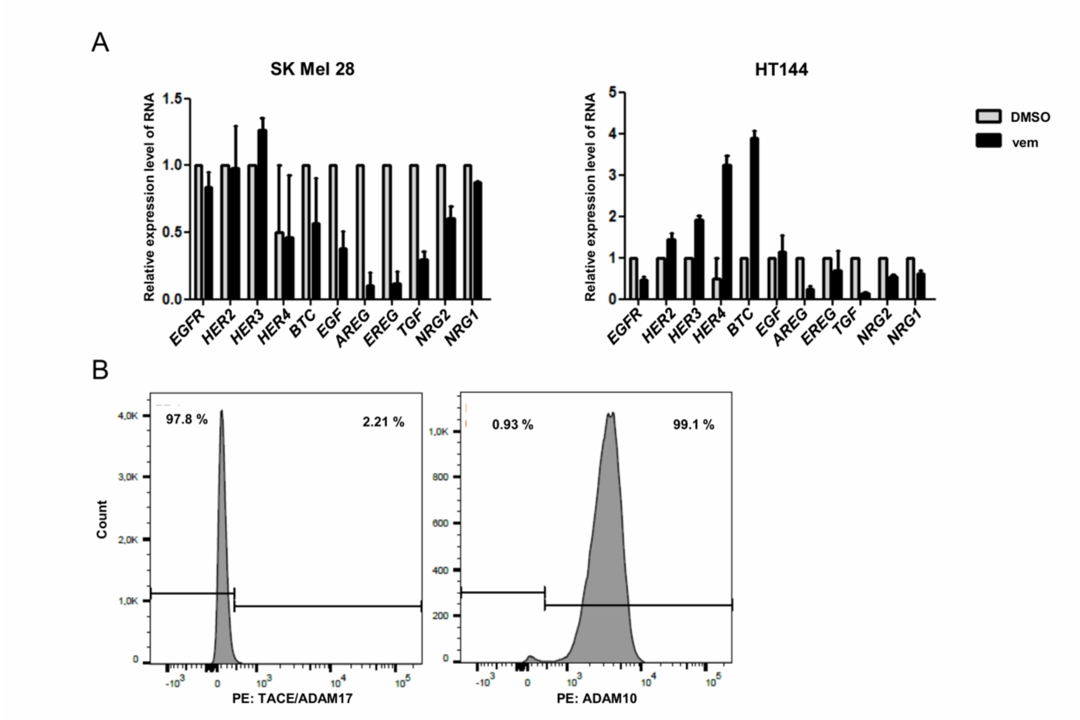

Figure S3: Expression of HER family members and their ligands. A: SK Mel 28 and HT144 cells were treated for 24h with vem (3 µM) or DMSO as a control. Relative expression levels of *HER* family members and their ligands determined by qPCR normalized to 18S. B: Flow cytometry analysis showing lack of TACE/ADAM17 but high expression of ADAM10 in SK Mel 28 cells.

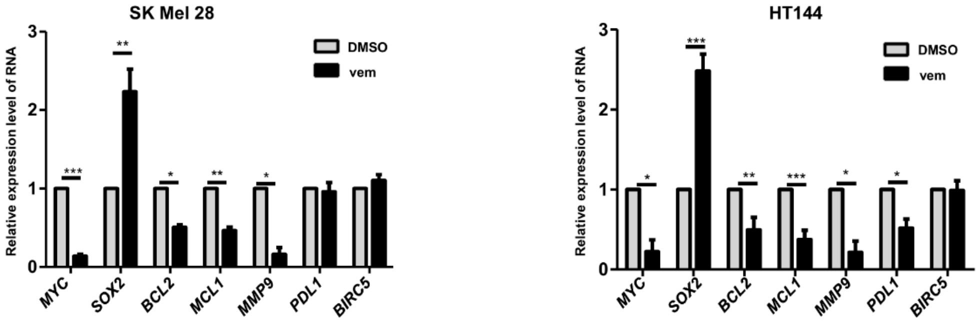

Figure S4: Expression of STAT3 target genes is changed after vem treatment. SK Mel 28 and HT144 cells were treated for 6h with vem (3 µM) or DMSO as a control. The relative expression of STAT3 target genes was analyzed by qPCR. ($p < 0.05$ *; $p < 0.01$ **; $p < 0.001$ ***).

Table S1. Combination index (CI) of A375 cells sensitive and resistant to vem. The CI was used to calculate if the drugs act synergistic or additive. A drug effect is considered synergistic if the CI is below 1. If the CI is not below 1 the effect is additive.

A375 res

drug1	*drug2*	*combination*	*d1*	*d2*	*Effect*	*CI*
A375.vem	*A375.gef*	*A375.vem+gef*	*20000*	*20000*	*0,40060315*	*0.01*
A375.vem	*A375.sap*	*A375.vem+.sap*	*20000*	*20000*	*0,51826159*	*0.07*
A375.vem+gef	*A375.sap*	*A375.vem+.gef+.sap*	*20000*	*20000*	*0,27092549*	*0.02*
A375.vem+.sap	*A375.gef*	*A375.vem+.gef+.sap*	*20000*	*20000*	*0,27092549*	*0.00*

A375

drug1	*drug2*	*combination*	*d1*	*d2*	*Effect*	*CI*
A375.vem	*A375.gef*	*A375.vem+gef*	*20000*	*20000*	*0,25557753*	*0.02*
A375.vem	*A375.sap*	*A375.vem+.sap*	*20000*	*20000*	*0,44621448*	*0.47*
A375.vem+gef	*A375.sap*	*A375.vem+.gef+.sap*	*20000*	*20000*	*0,16178206*	*0.05*
A375.vem+.sap	*A375.gef*	*A375.vem+.gef+.sap*	*20000*	*20000*	*0,16178206*	*0.01*
